# IGF1R- and ROR1-Specific CAR T Cells as a Potential Therapy for High Risk Sarcomas

**DOI:** 10.1371/journal.pone.0133152

**Published:** 2015-07-14

**Authors:** Xin Huang, Haein Park, Joseph Greene, James Pao, Erin Mulvey, Sophia X. Zhou, Catherine M. Albert, Fred Moy, Deepali Sachdev, Douglas Yee, Christoph Rader, Carl V. Hamby, David M. Loeb, Mitchell S. Cairo, Xianzheng Zhou

**Affiliations:** 1 Department of Pediatrics, Division of Hematology, Oncology and Stem Cell Transplantation, New York Medical College, Valhalla, NY, United States of America; 2 University of Minnesota College of Biological Sciences, Minneapolis, MN, United States of America; 3 New York Medical College School of Medicine, Valhalla, NY, United States of America; 4 Masonic Cancer Center, University of Minnesota, Minneapolis, MN, United States of America; 5 Division of Pediatric Oncology, Sidney Kimmel Comprehensive Cancer Center, Johns Hopkins University, Baltimore, MD, United States of America; 6 Department of Pathology, New York Medical College, Valhalla, NY, United States of America; 7 Department of Cancer Biology, The Scripps Research Institute, Jupiter, FL, United States of America; 8 Department of Molecular Therapeutics, The Scripps Research Institute, Jupiter, FL, United States of America; 9 Department of Microbiology and Immunology, New York Medical College, Valhalla, NY, United States of America; 10 Department of Cell Biology and Anatomy, New York Medical College, Valhalla, NY, United States of America; 11 Department of Medicine, New York Medical College, Valhalla, NY, United States of America; University of Navarra, SPAIN

## Abstract

Patients with metastatic or recurrent and refractory sarcomas have a dismal prognosis. Therefore, new targeted therapies are urgently needed. This study was designed to evaluate chimeric antigen receptor (CAR) T cells targeting the type I insulin-like growth factor receptor (IGF1R) or tyrosine kinase-like orphan receptor 1 (ROR1) molecules for their therapeutic potential against sarcomas. Here, we report that IGF1R (15/15) and ROR1 (11/15) were highly expressed in sarcoma cell lines including Ewing sarcoma, osteosarcoma, alveolar or embryonal rhabdomyosarcoma, and fibrosarcoma. IGF1R and ROR1 CAR T cells derived from eight healthy donors using the *Sleeping Beauty* (SB) transposon system were cytotoxic against sarcoma cells and produced high levels of IFN-γ, TNF-α and IL-13 in an antigen-specific manner. IGF1R and ROR1 CAR T cells generated from three sarcoma patients released significant amounts of IFN-γ in response to sarcoma stimulation. The adoptive transfer of IGF1R and ROR1 CAR T cells derived from a sarcoma patient significantly reduced tumor growth in pre-established, systemically disseminated and localized osteosarcoma xenograft models in NSG mice. Infusion of IGF1R and ROR1 CAR T cells also prolonged animal survival in a localized sarcoma model using NOD/scid mice. Our data indicate that both IGF1R and ROR1 can be effectively targeted by SB modified CAR T cells and that such CAR T cells may be useful in the treatment of high risk sarcoma patients.

## Introduction

Adoptive T-cell therapy (ACT) is a promising cancer treatment [1]. ACT including tumor infiltrating lymphocytes (TILs) or T cells engineered with tumor antigen-specific T cell receptors (TCRs) have achieved an objective response rate of approximately 70% in metastatic melanoma [2]. Recent Phase I clinical trials with CD19-targeted, 2^nd^ generation of chimeric antigen receptor (CAR) T cells containing 4-1BB signaling domain have shown a complete remission (CR) rate of >86% in pediatric and adult patients with relapsed/refractory acute lymphoblastic leukemia (ALL) [3]. In addition, CD19 CAR T cell therapy alone or in combination with hematopoietic stem cell transplantation also showed promise in adult patients with chronic lymphocytic leukemia (CLL) and ALL [4, 5]. Due to this high rate of efficacy, CD19 CAR T cells (CTL019) have received a breakthrough therapy designation from the FDA. Subsequently, CAR T cells have taken the lead as novel targeted cellular therapies for high risk, recurrent hematologic malignancies [6].

The encouraging results with CAR T cells in hematologic malignancies have spurred a growing interest in using this approach for solid tumors. CAR T cells targeting vascular endothelial growth factor receptor 2 (VEGFR2), epidermal growth factor receptor variant III (EGFRvIII), and mesothelin are being tested in patients with glioblastoma, pancreatic, ovarian and mesothelioma cancers [7]. In sarcomas, ACT with NY-ESO-1 TCR has demonstrated objective clinical responses in four of six patients with synovial cell sarcoma [8]. CAR targeted T-cell therapies in preclinical immunodeficient mouse models against GD2, IL-11Rα, HER2, and fetal acetylcholine receptor have shown specific cytotoxicity against Ewing sarcoma (EWS), neuroblastoma, osteosarcoma (OS) and rhabdomyosarcoma (RMS) [9–13]. A recent phase I/II clinical trial with HER2-CAR T cells (with CD28 signaling domain) in patients with recurrent/refractory HER2^+^ sarcoma demonstrated CAR-T cell persistence for 6 weeks without evident toxicities [14]. However, the clinical benefit of CAR T cells in patients with metastatic or recurrent/refractory sarcomas remains unknown.

Type I insulin-like growth factor receptor (IGF1R) is expressed in a wide range of solid tumors and hematologic malignancies [15, 16]. More importantly, IGF1R is necessary for the transforming ability of several oncogenes [17]. Recent clinical trials evaluating IGF1R-targeting monoclonal antibodies (mab) in patients with refractory EWS resulted in a modest overall response rate of 10–14% and only modest median progression-free survivals of less than 2 years [18–20]. While a randomized Phase II study testing the addition of the IGF1R mab ganitumab to chemotherapy in EWS is ongoing (NCT02306161), CAR T cells targeting IGF1R may be an alternative treatment for high risk patients with EWS and other sarcomas.

Overexpression of tyrosine kinase-like orphan receptor 1 (ROR1) has been documented in B-CLL, mantle cell lymphoma (MCL), breast cancer, B-ALL, lung adenocarcinoma, melanoma and ovarian cancer [21–30]. ROR1 has been shown to play a role in tumor cell migration and invasiveness and is not usually expressed in normal adult tissues except for B-cell precursors and adipose [22, 26, 31, 32]. Thus, ROR1 CAR T-cell therapy in sarcomas may yield an effective treatment with minimal off-target toxicity.

Here, we investigated CAR T-cell therapy targeting IGF1R and ROR1 in sarcomas. We demonstrated that *Sleeping Beauty* (SB) transposon-modified T cells with IGF1R- and ROR1-specific CARs were reactive against many types of sarcomas. We also showed that adoptive transfer of IGF1R- and ROR1-specific CAR T cells from a sarcoma patient significantly reduced tumor growth in pre-established, systemic and localized sarcoma xenograft models. Our results support future clinical trials in high risk sarcomas using SB modified CAR T cells targeting IGF1R and ROR1.

## Materials and Methods

### Ethics Statement

Human studies were conducted according to the principles expressed in the Declaration of Helsinki and approved by the Johns Hopkins University Institutional Review Board (IRB) under protocol NA_0028453. The IRB specifically approved this study. Collection of sarcoma patient’s peripheral blood was obtained after obtaining written informed consent which was approved by the Johns Hopkins University IRB. Animal studies were carried out in accordance with the recommendations in the Guide for Care and Use of Laboratory Animals of the National Institutes of Health and according to the University of Minnesota and New York Medical College Institutional Animal Care and Use Committee (IACUC). All animal studies were approved by the University of Minnesota IACUC under protocol 0901A57361, 1201A09281, 1312-31176A, and the New York Medical College IACUC under protocol 91-2-0912H. During *in vivo* experiment, animals were examined daily for a decrease in physical activity and other signs of disease. Severely ill animals (weight loss exceeding 20–25%, complete anorexia for 24 h, inability or extreme reluctance to stand which persists for 24 h, a lack of sustained purposeful response to gentle stimuli, and infection which fails to respond to antibiotic therapy) were euthanized by carbon dioxide. Recombinant DNA work was approved by the University of Minnesota and New York Medical College Institutional Biosafety Committee (IBC) under protocol 1006H83277 and 07-2012-8, respectively.

### Cell lines

K562 (erythroleukemia), Daudi (B-cell Burkitt lymphoma), MCF7 (breast cancer) cell lines, DB (B-cell lymphoma),EBV-LCL (EBV-transformed B lymphoblasts) and RPMI8226 (multiple myeloma) were used as control cells. K562 and Daudi cells were purchased from ATCC. DB and RPMI8226 cell lines were purchased from ATCC and provided by Dr. Mingqiang Ren (Georgia Regents University Cancer Center, Augusta, GA). MCF7 and EBV-LCL are from our laboratories [33–35]. A panel of human sarcoma cell lines including Ewing sarcomas (EWS): A673, EWS502, Rh1, SKNMC, TC32, TC71; alveolar rhabdomyosarcomas (ARMS): Rh18, Rh30, Rh41; embryonal rhabdomyosarcomas (ERMS): RD, Rh36; osteosarcomas (OS): OS17, U2OS, SaOS2 and fibrosarcomas (FS): HT1080 were tested in this study. Sarcoma cell lines Rh36, Rh41 and OS17 were provided by Dr. Peter Houghton (Nationwide Children's Hospital, Columbus, OH); lines Rh1, Rh18, Rh30, Rh41, U2OS, SaOS2, RD, and HT1080 were provided by Dr. David Largaespada (University of Minnesota, Minneapolis, MN); RD, U2OS, SaOs2 and HT1080 were purchased from ATCC; Rh1, Rh18, Rh31 and Rh41 were provided by Dr. Peter Houghton; and lines A673, EWS502, SKNMC, TC32 and TC71 were provided by Dr. Wen Luo and Dr. Stephen Lessnick (University of Utah, Salt Lake City, UT) [36, 37]. Information about Rh1, Rh18, Rh30, Rh36, Rh41 and OS17 can be found at the Pediatric Preclinical Testing Program website (http://gccri.uthscsa.edu/pptp/). SaOS2-fflucN, Rh30-fflucN and TC71-fflucN were lentivirally transduced cell lines expressing firefly luciferase and NGFR using pEhfflucmCNsin as previously described [38]. No authentication of these cell lines was done. These cell lines were maintained in RPMI 1640 medium supplemented with 10% heat-inactivated fetal bovine serum (FBS), 2 mM L-glutamine, 50 U/ml penicillin, and 50 μg/ml streptomycin. The A673 cell line was maintained in Dulbecco’s modified Eagle’s medium (DMEM) supplemented with 10% FBS and penicillin/streptomycin. R- cells,mouse fibroblast cell lines derived from a homozygous disruption of the *IGF1R* gene, and their human IGF1R expressing counterparts, R-/IGF1R cells,stably transfected with a plasmid encoding human IGF1R cDNA and hygromycin resistance [33], were provided by Dr. Deepali Sachdev and cultured in DMEM with 10% FBS, 50 U/ml penicillin, 50 μg/ml streptomycin, and 50 μg/ml G418 or 50μg/ml G418 plus 100 μg/ml hygromycin, respectively.

### Construction of SB transposons encoding IGF1R and ROR1 CARs

The SB transposons encoding IGF1R or ROR1 CARs were constructed using the terminal sequences previously described [38]. Anti-IGF1R and anti-ROR1 scFv sequences were derived from mouse mab 1H7 [34, 39] and 2A2 [24], respectively. The scFv, a CD8α hinge and transmembrane domain, and intracellular domains of 4-1BB and CD3ζ were assembled as IGF1R or ROR1 CARs based on the CTL019 construct provided by Dr. Carl June (University of Pennsylvania) [38].

### Human T cell gene transfer

Human T cell gene transfer was carried out using a Nucleofector device with the human T cell nucleofector kit (Lonza) as we previously described [38]. Peripheral blood mononuclear cells (PBMCs) were isolated from leukocytes which were purchased from the Memorial Blood Centers (St. Paul, MN) and New York Blood Center (New York, NY) or from 5–10 ml blood from 3 pediatric patients with OS, botyroid rhabdomyosarcoma (BRMS) and alveolar rhabdomyosarcoma (ARMS). PBMCs (5x10^6^) were transfected with SB CAR transposons (10 μg) mixed with SB100X transposase plasmid (10 μg) [38, 40], activated by anti-CD3/CD28 microbeads, and maintained in human T-cell medium consisting of RPMI-1640, 10% FBS, 10 mM HEPES, 2 mM L-glutamine, 50 μM β-mercaptoethanol, 50 U/ml penicillin, and 50 μg/ml streptomycin supplemented with IL-2 (50 IU/ml, Chiron Corp.) and IL-7 (10 μg/ml, R&D Systems). The activated T cells were sorted or selected for GFP^+^ cells by flow cytometry or zeocin (0.2 mg/ml, Invitrogen) selection and expanded every 10–14 days with anti-CD3/28 beads or OKT3 (Ortho Biotech) [38]. T cells cultured for 1–3 months were used for assays and were phenotypically effector memory T cells with variable ratios of CD4/CD8 subsets and stable expression of transgenes due to SB-mediated integration as we previously described [38, 41].

### Flow cytometric analysis

Alexa Fluor 647-conjugated F(ab’)_2_ fragments of goat anti-mouse IgG F(ab’)_2_ (anti-CAR, catalog #115-606-006) and Alexa Fluor 647-conjugated F(ab’)_2_ fragments of ChromPure goat IgG isotype control (015-600-006) were purchased from Jackson ImmunoResearch. Anti-human IGF1R-PE (clone 1H7), anti-human NGFR-PE (clone C40-1457) and isotype control mIgG1κ-PE (clone MOPC-21) were purchased from BD Biosciences. Anti-human ROR1-PE (clone 2A2) and mIgG1κ-PE (clone MOPC-21) isotype were purchased from BioLegend. Goat anti-human ROR1 polyclonal ab (catalog #AF2000), normal goat IgG control (AB-108-C) and allophycocyamin conjugated donkey anti-goat IgG (F0108) were purchased from RnD Systems. Flow cytometric analysis was performed on a BD FACSCaliber or Miltenyi MACSQuant cytometer, and data were analyzed with FlowJo software.

### Cytotoxicity and cytokine release assays

T cell cytotoxicity was carried out using a chromium (^51^Cr) release assay [38]. Cytokine release assays were performed by coculture of 1x10^5^ T cells with 2x10^4^ target cells per well in duplicate in 96-well flat-bottom plates. After 24 hr, supernatants were assayed using IFN-γ or TNF-α ELISA kits (Biolegend) and 4 or 16 human cytokine multiplex ELISA kits (Quansys Biosciences).

### *In vivo* anti-tumor assays

Breeding pairs of NOD.CB17-*Pkrdc* ^scid^/J (NOD/scid, stock number 001303) and NOD.Cg-*Prkdc* ^*scid*^ *IL2rg* ^*tm1WjI/SzJ*^ (NSG, stock number 005557) mice were purchased from the Jackson Laboratory (Bar Harbor, ME) and housed in the specific pathogen free facility with autoclaved cages,food and water. To establish a model of systemically disseminated sarcoma, 6–12 week old NSG mice with mixed genders were X ray-irradiated (2.5 Gy) on day -6 and inoculated intravenously (i.v.) with 7.5x10^5^ SaOS2-fflucN cells on day -5. On day -1, mice were examined for tumor engraftment by bioluminescent imaging (BLI) using the Xenogen-IVIS Imaging System [38]. Mice were randomly divided into three groups based on imaging intensity and sex. On day 0 and 3, three groups of tumor-bearing mice were infused i.v. twice on day 0 and day 3 with mock, IGF1R CAR (IGZ), and ROR1 CAR (RGZ) T cells (1x10^7^ per mouse) derived from sarcoma patient 1 as we previously reported in leukemia models [38]. Tumor BLI was performed on day 5, 11, 18, 25 and 32 after the first T-cell infusion. BLI was carried out under isoflurane anesthesia after intraperitoneal injection of D-luciferin (Xenogen, Hopkinton, MA). Images were collected and analyzed using the Xenogen-IVIS Imaging System. A constant region-of-interest (ROI) was drawn over the tumor region and the intensity of the signal measured as total photon flux normalized for exposure time and surface area, and expressed in units of photons/sec/cm^2^/steradian (p/sec/cm^2^/sr). To establish a localized sarcoma tumor model, 6–12 week old NOD/scid mice with mixed genders were irradiated (2.75 Gy, X-ray) on day -4 and inoculated intraperitoneally (i.p.) with 3x10^5^ SaOS2-fflucN on the following day. Mice were examined for tumor engraftment by BLI on day -1 and were randomly divided into four groups. On day 0, 2, and 4, three groups of tumor-bearing mice were injected i.p. with mock, IGZ and RGZ T cells (1x10^7^ per mouse), respectively, and one group was untreated. Tumor BLI was performed on day 6, 14, 21, 50 and 90 after the first T-cell infusion. Mice were then monitored for survival without imaging manipulations.

### Statistical analyses

All pairwise mean comparisons were ordinary *t*-tests adjusting for variance homogeneity as necessary and the Bonferroni adjustment for multiple mean comparisons was applied where appropriate following analysis of variance. Natural log transformation was utilized to stabilize the variance for the outcome variables. Kaplan-Meier survival curves were analyzed with the Mantel Haenzel test. Statistical analyses were conducted using SAS 9.1 software (Cary, NC) and NCSS 9.0 (Kaysville, UT). *P* values less than .05 were considered statistically significant.

## Results

### SB mediates coexpression of IGF1R or ROR1 CARs and GFP in primary T cells

**Fig 1:**
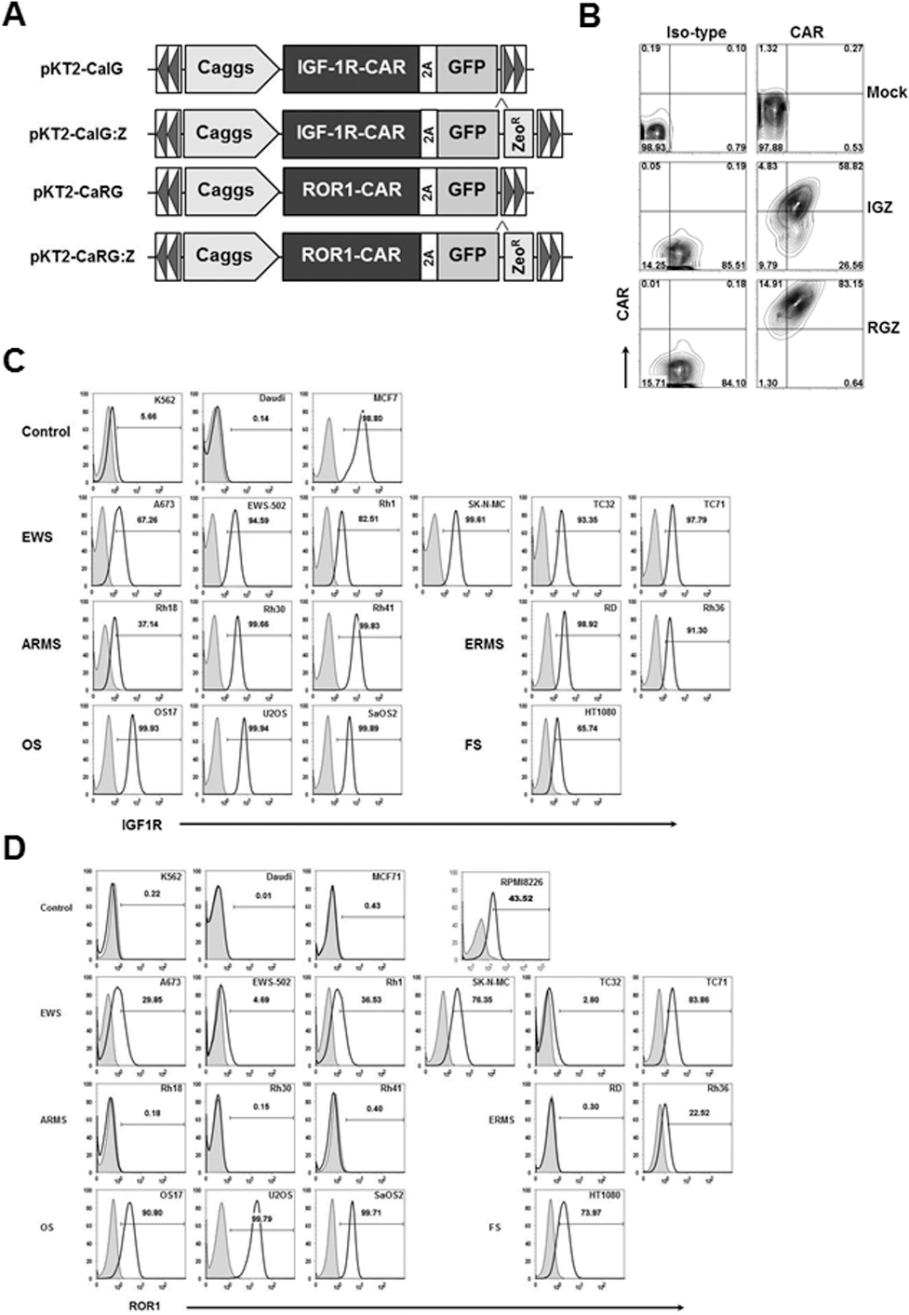
Cell surface expression of chimeric antigen receptor (CAR) and GFP after transfection and expression of IGF1R and ROR1 in a panel of sarcoma cell lines. (A) Schematic representation of the *Sleeping Beauty* (SB) transposons encoding IGF1R CAR and GFP (pKT2-CaIG), IGF1R and GFP:Zeocin (pKT2-CaIG:Z), ROR1 CAR and GFP (pKT2-CaRG), and ROR1 CAR and GFP:Zeocin (pKT2-CaRG:Z). The CAR contains the 4-1BB signaling domain (not shown). A Caggs promoter and a “self-cleaving” 2A peptide flanking the CAR sequence were used to regulate co-expression of CARs and *GFP* or *GFP-Zeocin* fusion in the SB transposon *trans* vectors, namely pKT2-CaIG, pKT2-CaRG, pKT2-CaIG:Z, and pKT2-CaRG:Z. (B) Expression of CAR and GFP in T cells derived from a sarcoma patient (Patient 1) after transfection of PBMCs with pKT2-CaIG:Z/pCMV-SB100X or pKT2-CaRG:Z/pCMV-SB100X or no DNA (as mock) and selection with zeocin. Similar data were obtained in T cells from other two patients with sarcomas and two healthy donors (data not shown). (C) Flow cytometric analysis of IGF1R expression in sarcoma cell lines including Ewing sarcoma (EWS), alveolar or embryonal rhabdomyosarcoma (ARMS or ERMS), osteosarcoma (OS), and fibrosarcoma (FS). K562 (erythroleukemia), Daudi (B-cell Burkitt lymphoma), MCF7 (breast cancer) cell lines were used as control. (D) Flow cytometric analysis of ROR1 expression in sarcoma cell lines. RPMI8226 (multiple myeloma) cell line was used as control. Similar data shown in (C) and (D) were obtained in at least three independent assays.

SB transposon constructs containing IGF1R and ROR1 CARs and GFP or GFP-Zeocin are shown in Fig 1A. PBMCs from at least 8 healthy donors and 3 pediatric sarcoma patients were nucleofected with SB100X transposase plus SB-IGF1R CAR (pKT2-CaIG and pKT2-CaIG:Z), SB-ROR1 CAR (pKT2-CaRG and pKT2-CaRG:Z), or without DNA as mock. After nucleofection and expansion, the sorted or zeocin-selected cells were analyzed for coexpression of both CARs and GFP. Greater than 58% of nucleofected T cells from patient 1 expressed CARs and GFP in both IGF1R CAR (IGZ) and ROR1 CAR (RGZ) T cell populations (Fig 1B). Mock T cells from the same patient were double negative for CARs and GFP. Similar levels of IGF1R or ROR1 CAR and GFP coexpression were observed in nucleofected PBMCs from two healthy donors and two sarcoma patients compared to SB modified T cells co-expressing CD19 CARs and CD20 as we previously reported (data not shown) [38]. These results confirm that SB can achieve a high level of coexpression of CARs and a reporter gene in primary T cells derived from sarcoma patients and healthy donors [38].

### IGF1R and ROR1 are broadly expressed in sarcomas

We have previously shown that a chimeric scFv against IGF1R can downregulate IGF1R in breast cancer cells, a general mechanism in antibody resistance [33]. Thus, our hypothesis is that IGF1R CAR T cells can kill IGF1R^+^ tumor cells even with a low level of surface expression. To test our hypothesis, we first evaluated IGF1R surface expression in a panel of sarcoma cell lines using flow cytometry. We found that all sarcoma lines tested including 6 EWS (A673, EWS502, Rh1, SKNMC, TC32, TC71), 3 ARMS (Rh18, Rh30, Rh41), 2 ERMS (RD, Rh36), 3 OS (OS17, U2OS, SaOS2) and 1 FS (HT1080) were IGFR1^+^ (Fig 1C). The MCF7 breast cancer line had a high level of IGFR1 expression while the K562 erythroleukemia and Daudi lymphoma lines had a low or no expression of IGF1R, respectively. Note that most sarcoma cell lines appear to express low levels of IGF1R on the surface.

ROR1 is identified as a highly expressed gene in B-cell CLL, but not normal B cells, suggesting it may serve as a tumor-specific target for therapy [21, 24]. In addition, ROR1-specific CAR T cells from healthy donors or CLL patients conferred specific recognition of primary B-CLL and MCL, including chemotherapy resistant tumor cells, but not mature normal B cells [32]. Our group also constructed a ROR1 CAR in the SB transposon and showed unexpectedly that ROR1 CAR T cells were cytotoxic against both RPMI8226 multiple myeloma and SaOS2 OS cell lines (data not shown). This experiment prompted us to examine ROR1 expression in sarcoma lines. Fig 1D demonstrated that all 6 EWS, 3 OS and 1 FS cell lines were ROR1^+^ whereas 3 ARMS were ROR1^-^ and 1 of 2 ERMS lines appeared to be ROR1^+^. Control K562, Daudi, and MCF7 cells were ROR1^-^, whereas a positive control RPMI8226 line was ROR1^+^ [32]. These results indicate that ROR1 could serve as a potential target for EWS, OS and ERMS tumors.

### IGF1R and ROR1 CAR T cells kill sarcomas selectively

**Fig 2:**
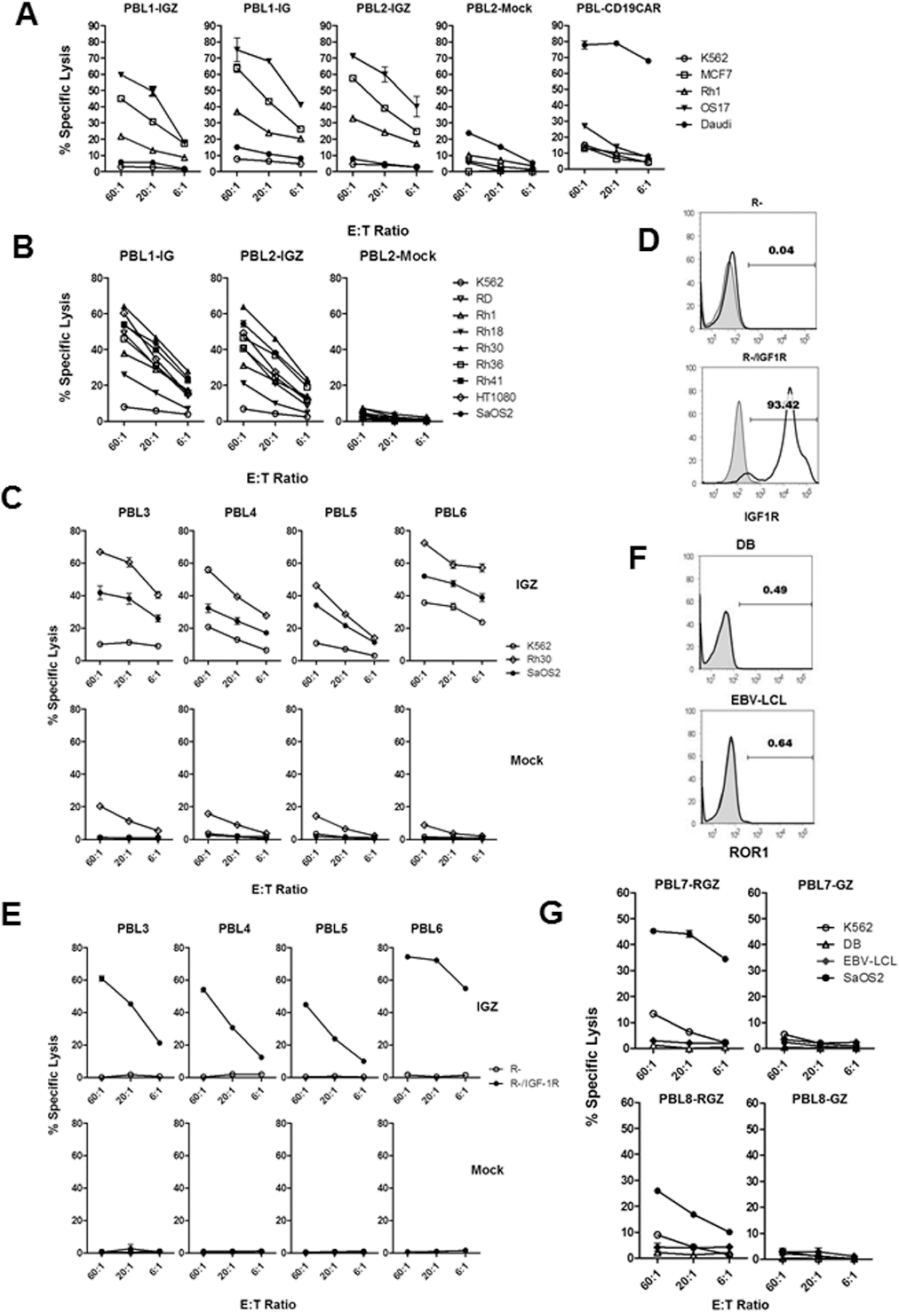
*In vitro* anti-sarcoma cytotoxicity of IGF1R and ROR1 CAR T cells. (A) Cytotoxicity against IGF1R^+^ target cells including sarcoma cell lines by SB modified IGF1R CAR T cells derived from two healthy donors. PBL1-IGZ, PBL1-IG, PBL2-IGZ and PBL2-mock T cells were generated by transfection of PBMCs derived from two healthy donors (PBL1 and PBL2) using pKT2-CaIG:Z or pKT2-CaIG plus pCMV-SB100X plasmids or without DNA. PBL-CD19CAR T cells were used as control. PBL1-IGZ: Rh1 vs K562 and OS17 vs K562, *p* < 0.001 at all 3 E/T ratios. PBL1-IG: Rh1 vs K562 and OS17 vs K562, *p* < 0.01. PBL2-IGZ: Rh1 vs K562, *p* < 0.001, OS17 vs K562, *p* < 0.01. Killing of Rh1 and OS17 in each CAR T cell group was also significant compared to the corresponding tumor cells in mock T cell group (*p* < 0.05). (B) Cytotoxicity of IGF1R CAR T cells against a panel of sarcoma cell lines. Comparisons in PBL1-IGZ and PBL2-IGZ were conducted between each sarcoma line and K562, *P* = 0.0001 at all E/T ratios. (C) Cytotoxicity against sarcoma cells by IGF1R CAR T cells derived from four more healthy donors but not by mock T cells. Comparisons in PBL3-IGZ, PBL4-IGZ, PBL5-IGZ and PBL6-IGZ were conducted between Rh30 vs K562 and SaOS2 vs K562, *P* = 0.0001 at all E/T ratios.(D) Expression of human IGF1R in R- transfected cell line confirmed by flow cytometry. (E) Specific cytotoxicity against human IGF1R transfected cell line by IGF1R CAR T cells. Comparisons in PBL3-IGZ, PBL4-IGZ, PBL5-IGZ and PBL6-IGZ were conducted between R- and R-/IGF1R, *P* = 0.0001 at all E/T ratios. (F) Expression of ROR1 in DB and RBV-LCL cell lines. ROR1^-^ K562 and ROR1^+^ RPMI8226 were used as control (data not shown). (G) Cytotoxicity against ROR1^+^ target cells including a sarcoma cell line by ROR1 CAR T cells derived from two healthy donors. Comparisons in PBL7-RGZ, PBL7-GZ, PBL8-RGZ and PBL8-GZ were conducted between each target cell and K562 at all E/T ratios. PBL7-RGZ: SaOS2 vs K562, *p* = 0.0001, 0.0008, 0.0001 (E/T of 60:1, 20:1 and 6:1). PBL7-GZ: SaOS2 vs K562, *p* = 0.0221, 0.0762, 0.1121. PBL8-RGZ: SaOS2 vs K562, *p* = 0.0001, 0.0009, 0.0041. PBL8-GZ: SAOS2 vs K562, *p* = 0.2585, 0.8439, 0.9298. *P* values for DB vs K562 and EBV-LCL vs K562 were not shown. All cytotoxicity data shown are mean ± S.E. of triplicates.

Next, we determined if SB modified IGF1R and ROR1 CAR T cells kill sarcomas in an antigen-specific manner. We initially generated IGF1R CAR T cells from two healthy donors with two SB-IGF1R CAR constructs (pKT2-CaIG and pKT2-CaIG:Z). ^51^Cr-release assays showed that IGF1R CAR T cells from PBL1 (PBL1-IGZ and PBL1-IG) and PBL2 (PBL2-IGZ) significantly killed both Rh1 and OS17 sarcoma cells as well as the IGF1R^+^ MCF7 breast cancer line at all effector/target ratios of 60:1, 20:1 and 6:1 compared to K562 and Daudi control cells (Fig 2A, *p* < 0.01 by Pairwise t-test). IGF1R^-^ K562 and Daudi cells were not killed (at 20:1 and 6:1, *p* >0.05). As a control, SB modified CD19 CAR T cells only killed CD19^+^ Daudi but not sarcoma cells. Unmodified mock T cells did not kill Rh1, OS17, and MCF7 compared to IGF1R CAR T cells (PBL1-IGZ, PBL1-IG and PBL2-IGZ) at all 3 E/T ratios (*p* >0.05).

IGF1R CAR T cells from two donors also killed a panel of sarcomas lines including 1 EWS (Rh1), 1 OS (SaOS2), 1 FS (HT1080), 3 ARMS (Rh18, Rh30, Rh41) and 2 ERMS (RD, Rh36) (vs K562, *p* = 0.001, Fig 2B). Again, unmodified mock T cells were not cytotoxic to any sarcoma lines.

We then generated IGF1R CAR T cells from 4 more healthy donors (PBL3, 4, 5, and 6) and evaluated their cytotoxic function. IGF1R CAR T cells (IGZ) from those 4 donors were cytotoxic to Rh30 and SaOS2 sarcoma cells (vs K562, *p* = 0.0001, Fig 2C). A low level of killing of K562 cells was observed in PBL4 and PBL6 CAR T cells as well as in all mock T cells from PBL4 to PBL6 which might be related to nonantigen specific cytotoxicity (Fig 2C). To further demonstrate IGF1R CAR T cell specificity, we tested their cytotoxicity against mouse fibroblast cells (R-) derived from IGF1R deficient mice and human IGF1R cDNA transfected R- cells (R-/IGF1R) which was confirmed by flow cytometry (Fig 2D). IGF1R CAR T cells but not mock T cells only killed R-/IGF1R but not R- cells (p = 0.0001), as shown in Fig 2E, suggesting that IGF1R CAR T cells are highly specific in targeting IGF1R.

We also evaluated ROR1 CAR T cell cytotoxicity against sarcoma cells using ^51^Cr-release assays. PBMCs from 2 healthy donors (PBL7 and PBL8) were engineered with SB ROR1 CAR (pKT2-CaRG:Z) and control pKT2-CaG:Z (Fig 1A). ROR1 CAR T cells (PBL7-RGZ and PBL8-RGZ) significantly killed ROR1^+^ SaOS2, but not ROR1^-^ DB and EBV-LCL cells, which were negative for ROR1 by flow cytometry (Fig 2F) (*p* < 0.01 at E/T of 20:1 and 6:1, Fig 2G, a low level of non-specific cytotoxicity against K562 was noted). Modified control T cells (PBL7-GZ and PBL8-GZ) did not kill sarcoma and other cells (*p* > 0.05). Altogether, these results firmly demonstrate that both IGF1R and ROR1 CAR T cells are cytotoxic to antigen^+^ sarcoma cells *in vitro*.

### IGF1R and ROR1 CAR T cells produce cytokines in response to sarcoma stimulation

**Fig 3:**
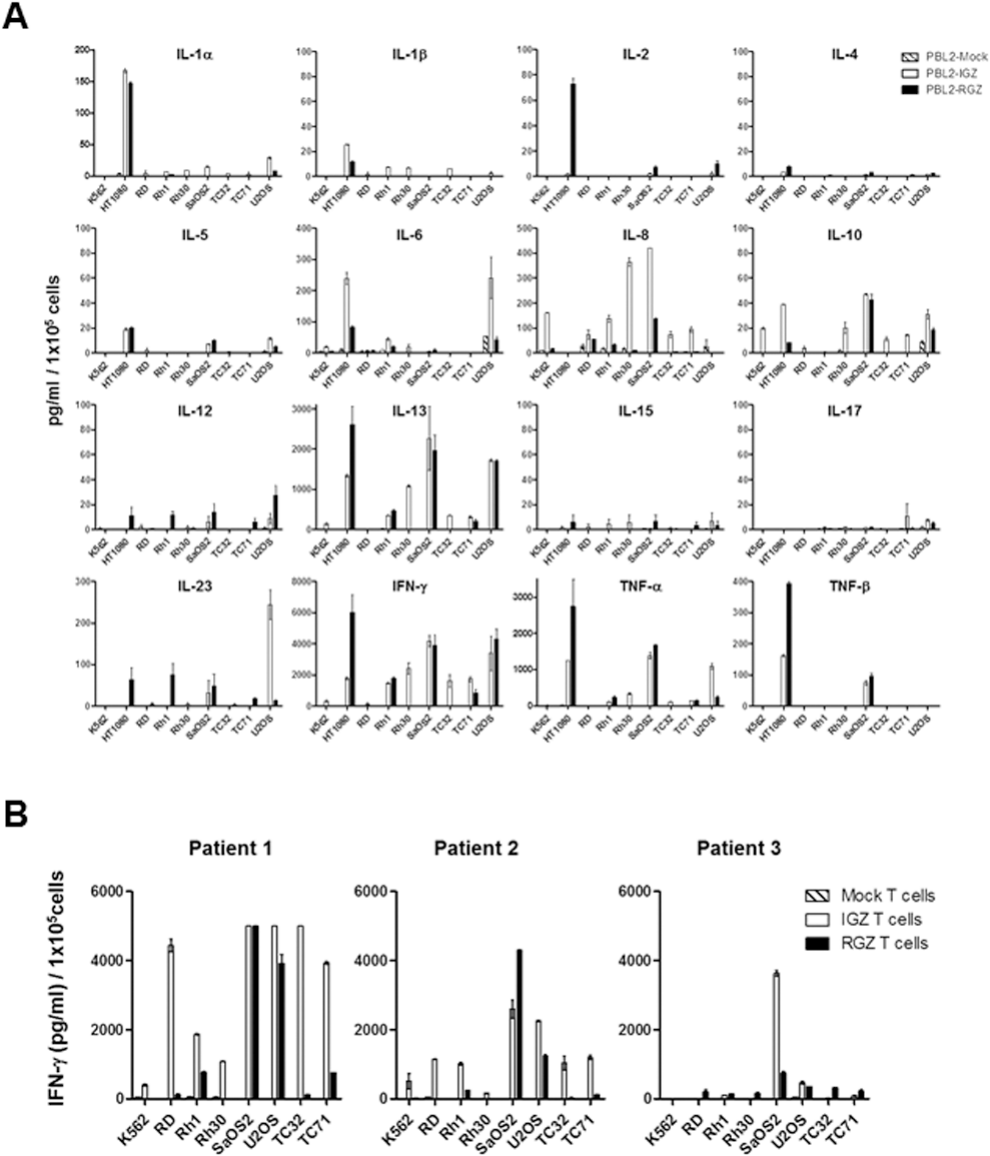
Cytokine profiling of IGF1R and ROR1 CAR T cells in response to sarcoma stimulation. (A) Significant production of IFN-γ, TNF-α, and IL-13 by IGF1R and ROR1 CAR T cells derived from a healthy donor. Data shown are mean ± S.E. of duplicates. One out of two representative data is shown. (B) Antigen-specific production of IFN-γ by IGF1R and ROR1 CAR T cells derived from three sarcoma patients. Data shown are mean ± S.E. of duplicates.

Both IGF1R CAR and ROR1 CAR T cells derived from a healthy donor produced high-levels of Th1 cytokines including IFN-γ and TNF-α in an antigen-specific manner (Fig 3A). For example, HT1080, Rh1, SaOS2, TC71 and U2OS cells (IGF1R^+^ROR1^+^) were recognized by both CAR T cells whereas RD and Rh30 cells (IGF1R^+^ROR1^-^), and TC32 (IGF1R^+^ROR1^+/-^) were only recognized by IGF1R CAR T cells but not ROR1 CAR T cells. Also, both CAR T cells produced high amounts of IL-13 Th2 cytokine but not IL-4 and IL-10. IGF1R CAR T cells produced higher amounts of IL-6 and IL-18 than ROR1 CAR T cells in response to certain sarcoma lines. There was no significant production of IL-1α, IL-1β, IL-2, IL-5, IL-12, IL-15, IL-17, and IL-23 by both CAR T cells. Mock T cells or CAR T cells co-cultured with ROR1^-^ K562 cells did not release cytokines whereas IGF1R CAR T cells co-cultured with IGF1R^+/-^ K562 cells produced a low level of cytokines compared to IGF1R^+^ sarcomas. In addition, both IGF1R and ROR1 CAR T cells but not mock T cells derived from 3 patients with sarcoma released significant amounts of IFN-γ in response to specific sarcoma antigen stimulation (Fig 3B).

### IGF1R and ROR1 CAR T cells can mount anti-sarcoma responses *in vivo*

We employed a time-dynamic bioluminescent imaging (BLI) technique in live mice to assess the anti-sarcoma effect of SB-engineered IGF1R and ROR1 CAR T cells. Three sarcoma lines (Rh30, SaOS2 and TC71) were transduced with a lentiviral vector carrying firefly luciferase (ffluc) and nerve growth factor receptor (NGFR) transgenes (S1 Fig). The transduced sarcoma lines (Rh30-fflucN, SaOS2-fflucN, TC71-fflucN) were tested to confirm NGFR expression and were found to express a high-level of lucifearse activity (S1 Fig). The transduced sarcoma lines were recognized as efficiently as the parent lines by patient 1 IGF1R and ROR1 CAR T cells (S1A Fig). Because SaOS2-fflucN cell line was recognized by both CAR T cells in terms of cytotoxicity and IFN-γ release (Figs 2B, 2C, 2G, 3A, 3B and S1B), we used this line for adoptive T cell transfer experiments.

**Fig 4:**
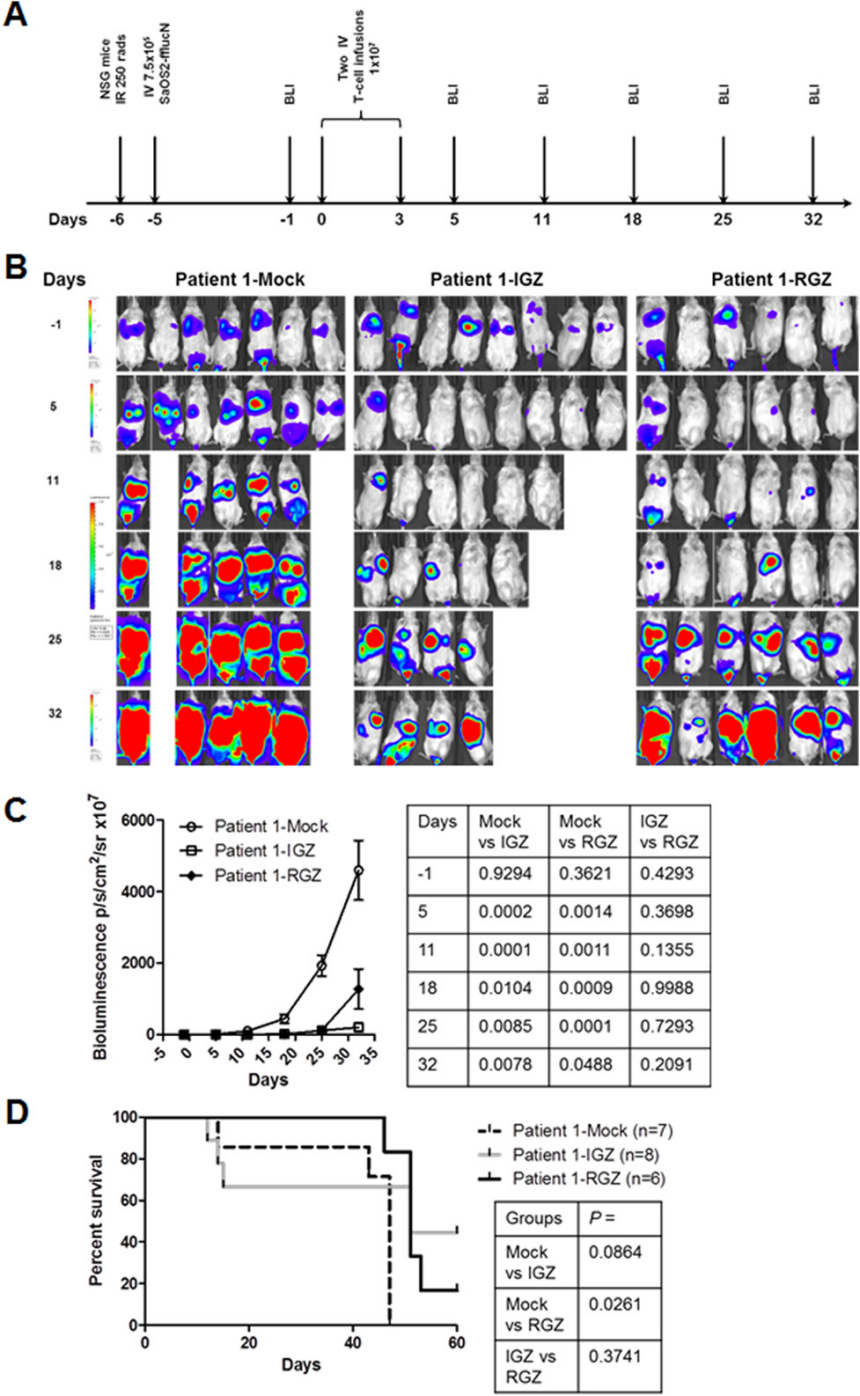
*In vivo* anti-sarcoma activity of IGF1R and ROR1 CAR T cells in a disseminated mouse model. (A) The experimental schedule of tumor cell injection, CAR T cell infusion and BLI monitoring. Prior to testing, all mice displayed normal healthy status. B) Bioluminescent imaging (BLI) of tumor growth in NSG mice (three groups, n = 6–8 each) treated with a sarcoma patient derived T cells expressing IGF1R CAR (IGZ), ROR1 CAR (RGZ) or mock T cells. SaOS2-fflucN cells were transduced with a lentiviral vector expressing humanized firefly luciferase and truncated nerve growth factor receptor (NGFR). Two mice in the mock group died of tumor progression on day 8. Four mice in IGZ group died of unknown causes on day 8, 9, 16 and 24, probably due to cytokine storms. (C) Bioluminescent intensity of the mice treated with the T cells. All *p* values were shown in the right panel table and were verified independently. (D) Animal survival after T-cell therapy. All *p* values were determined using Mantel-Haenszel logrank test and shown in the right panel table. The *p* values were independently confirmed. Note that *p* > 0.05 between mock vs IGZ was likely due to a small sample size.

For a disseminated sarcoma model, NSG mice were i.v. injected with SaOS2-fflucN cells after irradiation (Fig 4A). The administration of 2 infusions of either IGF1R CAR or ROR1 CAR T cells derived from patient 1 into tumor-bearing mice significantly suppressed sarcoma growth at days 5, 11, 18, 25 and 32 post T-cell infusions compared to mock T-cell treated mice (p ≤ 0.01 at days 5, 11, 18, 25; p <0.01 at day 32 for IGZ; *p* < 0.05 at day 32 for RGZ; IGZ vs RGZ, *p* > 0.05 at all day points, Fig 4B and 4C). A significant survival advantage was observed in ROR1 CAR but not IGF1R CAR T cell groups vs mock T cell control group (*p* = 0.0261 and *p* = 0.0864, Fig 4D). Recurrent tumors were recovered from both CAR T cell treated mice and confirmed that no significant loss of expression of IGF1R and ROR1 antigens and NGFR transgene occurred (S2 Fig). These experiments demonstrate that SB-engineered IGF1R and ROR1 CAR T cells from a sarcoma patient can function as antitumor effector cells *in vivo*.

**Fig 5:**
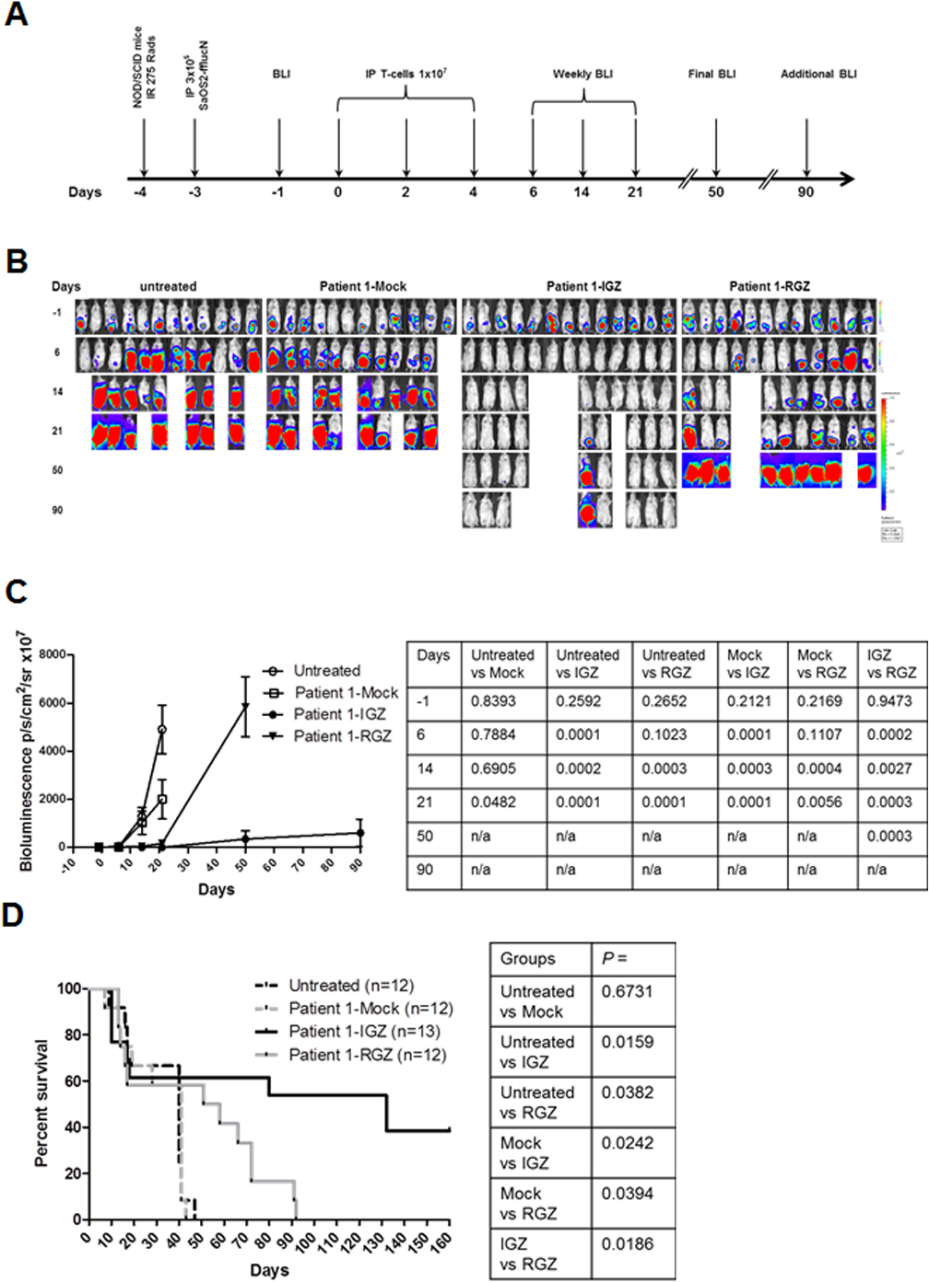
*In vivo* anti-sarcoma response by IGF1R and ROR1 CAR T cells in a localized tumor mouse model. (A) The experimental schedule of tumor cell injection, CAR T cell infusion and BLI monitoring. Prior to testing, all mice displayed normal healthy status. (B) Bioluminescent imaging (BLI) of tumor growth in NOD/SCID mice (four groups, n = 12–13 each) treated with a sarcoma patient derived T cells expressing IGF1R CAR (IGZ), ROR1 CAR (RGZ) or mock T cells. One group mice were untreated. Two mice in the untreated group died of tumor progression on day 13 and 20 and the other two mice died of unknown causes on day 12 and 13. Three mice in the mock group died of tumor progression on day 7, 12 and 19 andone died of unknown causes on day 13. Three mice in the IGZ group died of unknown causes on day 10 and two died of unknown courses on day 17 and 18. Two mice in the RZG group died of tumor progression on day 12 and 50 andone died of unknown causes on day 13. (C) Bioluminescent intensity of the mice treated with the T cells. All *p* values were shown in the right panel table and were verified independently. (D) Animal survival after T-cell therapy. All *p* values were determined by the Mantel-Haenszel logrank test and are shown in the right panel table.

For a local sarcoma model, NOD-scid mice were injected i.p. with SaOS2-fflucN (Fig 5A). The administration of 3 infusions IGF1R CAR T cells derived from patient 1 also significantly inhibited tumor growth compared to untreated group at days 6, 14 and 21 post T-cell infusions (*p* < 0.001, Fig 5B and 5C). Infusions of ROR1 CAR T cells did not observe immediate tumor reduction at day 6 compared to untreated and mock treated groups (*p* > 0.05). Significant anti-sarcoma activity was evident at days 14 and 21 by ROR1 CAR T cells (*p* < 0.01). In addition, IGF1R CAR and ROR1 CAR T cell treated animals survived up to a time point between day 50 and day 90, and day 50 and day 150, respectively (p < 0.05 compared to untreated and mock, Fig 5D). It seems that IGF1R CAR T cells were more potent than ROR1 CAR T cells in suppressing tumor growth in this localized model (*p* < 0.01, Fig 5B and 5C) and extending survival as up to 40% of the treated mice by IGF1R CAR T cells survived to at least until at day 160 whereas no ROR1 CAR T cell treated mice survived up to day 90 (*p* = 0.0186). Altogether, these results demonstrate that SB engineered IGF1R and ROR1 CAR T cells derived from a sarcoma patient can suppress sarcoma growth *in vivo*.

### Potential on-target toxicity by IGF1R and ROR1 CAR T cells

Although there have been few major toxicities during early phase trials of anti-IGF1R mabs, expression of IGF1R in normal tissues raises a concern about the potential for toxicity by IGF1R CAR T cells [42]. We examined IGF1R and ROR1 expression in PBMCs derived from three healthy donors and found that both lymphocytes and monocytes expressed a low level of IGF1R by flow cytometric analysis. Expression of ROR1 in both populations was not evident (S3 Fig). Co-culture of PBMCs with IGF1R and ROR1 CAR T cells derived from a sarcoma patient and a healthy donor revealed a low level of recognition of PBMCs by IGF1R CAR T cells but not ROR1 CAR T cells compared to sarcoma cells. These results suggest that targeting IGF1R but not ROR1 by CAR T cells has the potential of a low level of on-target toxicity.

## Discussion

This report presents data that support three novel findings. First, ROR1 is expressed in many types of sarcoma including EWS, OS and RMS, suggesting that ROR1 could be used as a therapeutic target in sarcomas. Second, SB modified CAR T cells derived from healthy donors and sarcoma patients targeting IGF1R or ROR1 displayed a specific cytotoxicity and released predominantly IFN-γ, TNF-α and IL-13 cytokines against sarcomas *in vitro*. Third, Both IGF1R and ROR1 CAR T cells derived from a sarcoma patient can significantly suppress sarcoma growth in both localized and disseminated pre-established sarcoma xenograft models. Moreover, adoptive transfer of IGF1R and ROR1 CAR T cells also demonstrated a prolonged survival benefit in a localized sarcoma model. Our data indicate that IGF1R and ROR1 CAR T cells could be used as a potential therapy for high risk sarcomas.

IGF1R is a tetrameric transmembrane receptor tyrosine kinase. Binding of ligand to IGF1R α subunits leads to β subunit autophosphorylation and recruitment of adapter proteins, ultimately leading to activation of signaling cascades that result in proliferation, survival, transformation, metastasis, and angiogenesis [17, 42]. IGF1R has been reported to be expressed in various hematologic and solid tumors including multiple myeloma, leukemia, lymphoma, breast, prostate, lung, colon, thyroid, renal, adrenal cancer, retinoblastoma, and sarcoma [16]. The role of IGF1R in pediatric cancers including EWS, ARMS, OS, synovial sarcoma, Wilms tumor, neuroblastoma, glioblastoma and medulloblastoma, has also been documented [43]. Therefore, successful targeting IGF1R has a broad impact on cancer treatment [17, 42, 43].

In addition to IGF1R inhibitors and mabs, IGF1R has been considered to be immunogenic and tested as a cancer vaccine. IGF1R-specific IgG antibodies were significantly elevated in early stage breast cancer patients. The magnitude of Th2 immunity against predicted T-helper epitopes derived from the IGF1R was also greater in breast cancer patients [44]. Furthermore, a multiantigen vaccine targeting Neu, IGFBP-2 and IGF1R was highly effective at preventing tumor progression in mice with preinvasive breast disease, whereas an individual vaccine was partially effective [45]. These results suggest that IGF1R could be targeted as a breast cancer antigen.

Despite promising preclinical data and a strong rationale for targeting IGF1R, clinical efficacy with anti-IGF1R mabs as single agents or in combination with chemotherapy has been disappointing in EWS, lung cancer and other diseases [18–20, 46, 47]. Several potential mechanisms exist by which tumors are resistant to IGF1R mabs, including downregulation of IGF1R, upregulation of insulin receptor (IR)-A and IGF-I and IGF-II, activation of MAPK/ERK or mTOR pathways, IGF1R-HER2 heterodimerization, and expression of other growth factor receptors [42, 47].

Targeting IGF1R by CAR T cells may represent a potential curative therapy for IGF1R^+^ cancer by providing several advantages over antibodies. First, they directly and specifically kill IGF1R^+^ cancer cells even at a low level of surface expression as CD8^+^ CTLs are capable of responding to a single antigen molecule [48]. Second, they produce TNF-α and IFN-γ cytokines, which subsequently mediate bystander elimination of stromal cells and vasculature within the tumor microenvironment [49]. Third, they persist for months and years, and proliferate *in vivo* as it has been shown in recent clinical trials with CD19 CARs containing 4-1BB or CD28 signaling domains [1, 3–5].

Expression of IGF1R in normal tissues raises a concern about the potential for toxicity by IGF1R targeted therapies [42]. We found that both lymphocytes and monocytes expressed lower levels of cell surface IGF1R, resulting a low level of recognition by IGF1R CAR T cells compared to sarcoma cell lines (S3 Fig). Systemic evaluation of on-target toxicity by IGF1R CAR T cells in canine sarcoma models will be tested as IGF1R CAR-derived 1H7 mab cross reacted with canine osteosarcoma lines (Park et al., unpublished data). In addition, several strategies could be developed to reduce on target toxicity by IGF1R CAR T cells, including co-expression of a suicide gene, use of mRNA transfection, and inclusion of PD-1- and CTLA-4-based inhibitory CARs (iCARs) [50].

We have also identified ROR1 as a potential antigen in the majority of EWS and OS, and a subset of RMS. ROR1 appears to be an attractive therapeutic target in sarcomas because of its limited expression in normal adult tissues. Importantly, ROR1 may act as a survival factor for sarcoma cells although its function in sarcoma is not known. ROR1 belongs to a large family of cell surface receptor tyrosine kinases. Upon ligand binding, ROR1 dimerizes and recruits canonical and non-canonical signaling pathways for cell survival and invasion [51].

Our preclinical data suggest that IGF1R CAR T cells mounted more potent anti-sarcoma activity than ROR1 CAR T cells *in vivo* (Figs 4C, 5C and 5D). This difference cannot readily be explained by CAR expression levels, antigen density on the tumor cell, T-cell growth and survival, T-cell cytotoxicity and cytokine production. A relatively higher affinity of IGF1R CAR (1H7 scFV-Fc monovalent, 1.0 x 10^−8^ M) than ROR1 CAR (2A2 scFV-IgG, 3.26 x 10^−8^ M) might contribute to this difference. Increasing ROR1 CAR affinity by substituting the 2A2 clone with the R12 clone (Fab, 0.56 x 10^−9^ M, 58 fold higher affinity than the 2A2 clone) could be considered to enhance ROR1 CAR T cell potency [24, 52]. It has been recently demonstrated that increasing affinity of ROR1-CARs could have significant antitumor efficacy *in vitro* and *in vivo* [52].

In conclusion, our results have demonstrated *in vitro* and *in vivo* anti-sarcoma activity of IGF1R and ROR1 CAR T cells. IGF1R and ROR1 CAR T cells may represent a new treatment option for patients with metastatic or relapsed/refractory sarcomas. Our future work will be focused on assessment of on-target toxicity by IGF1R CAR T cells in animal models and reexamination of ROR1 expression in adult normal tissues although ROR1 CAR T cells have recently shown to be safe in nonhuman primates [53].

## Supporting Information

S1 ARRIVE Checklist (PDF)

S1 Fig Recognition of lentiviral transduced sarcoma cell lines expressing humanized firefly luciferase by IGF1R and ROR1 CAR T cells. (A) Verification of lentiviral transduced sarcoma cell lines Rh30, SaOS2 and TC71 expressing humanized firefly luciferase and NGFR compared to their parental lines. (B) Unaltered recognition of lentiviral transduced sarcoma cell lines by IGF1R and ROR1 CAR T cells revealed by IFN-γ release assays. Data shown are mean ± S.E. of duplicates.
(TIF)

S2 Fig *Ex vivo* analysis of antigen expression in recurrent sarcoma after IGF1R and ROR1 CAR T cell infusion in a systemically disseminated sarcoma model. Tumors were dissected from moribund mice # 7, 8 and 19 after IGF1R and ROR1 CAR infusion, and digested into single cell suspension using DNase I (1 mg/mL) and collagenase (2 mg/mL) at 37°C for 1 hr.
(TIF)

S3 Fig Cell surface expression of IGF1R and ROR1 in PBMCs and impact on CAR T cell recognition. IGF1R and ROR1 expression in PBMCs derived from 3 healthy donors (PBL9-11) and gated on lymphocytes (A) and monocytes (B). (C) and (D) TNF-α release assays of IGF1R and ROR1 CAR T cells after co-culture with PBMCs derived from 3 healthy donors (PBL12-14). Data shown are mean ± S.E. of duplicates.
(TIF)
