# Supplementary figures and images for: IGF1R- and ROR1-Specific CAR T Cells as a Potential Therapy for High Risk Sarcomas
Source: PLoS One. 2015 Jul 14;10(7):e0133152. doi: 10.1371/journal.pone.0133152 (PMC4501840; doi:10.1371/journal.pone.0133152)

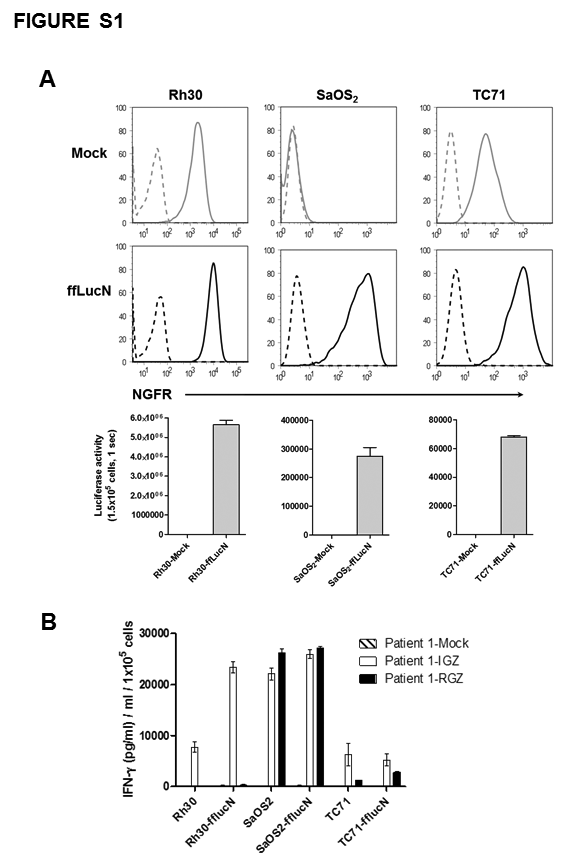

Supplement: S1 Fig — (A) Verification of lentiviral transduced sarcoma cell lines Rh30, SaOS2 and TC71 expressing humanized firefly luciferase and NGFR compared to their parental lines. (B) Unaltered recognition of lentiviral transduced sarcoma cell lines by IGF1R and ROR1 CAR T cells revealed by IFN-γ release assays. Data shown are mean ± S.E. of duplicates. (TIF) [file pone.0133152.s002.tif]

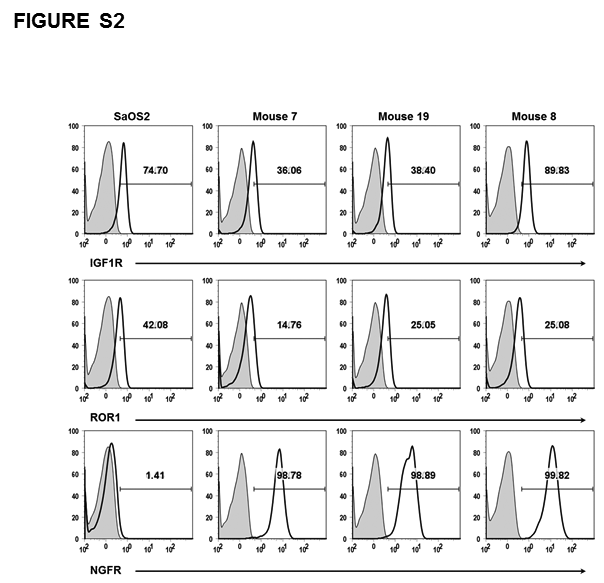

Supplement: S2 Fig — Tumors were dissected from moribund mice # 7, 8 and 19 after IGF1R and ROR1 CAR infusion, and digested into single cell suspension using DNase I (1 mg/mL) and collagenase (2 mg/mL) at 37°C for 1 hr. (TIF) [file pone.0133152.s003.tif]

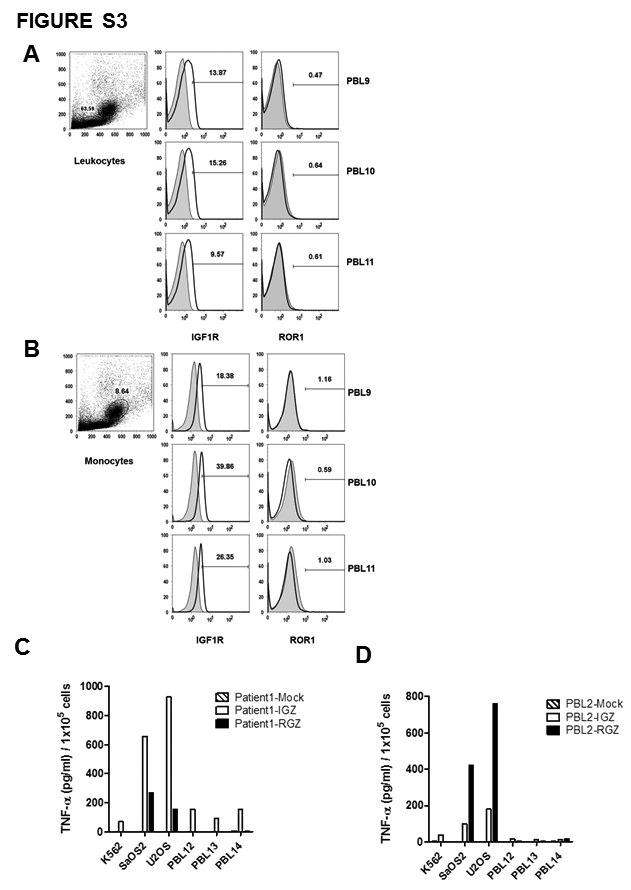

Supplement: S3 Fig — IGF1R and ROR1 expression in PBMCs derived from 3 healthy donors (PBL9-11) and gated on lymphocytes (A) and monocytes (B). (C) and (D) TNF-α release assays of IGF1R and ROR1 CAR T cells after co-culture with PBMCs derived from 3 healthy donors (PBL12-14). Data shown are mean ± S.E. of duplicates. (TIF) [file pone.0133152.s004.tif]
